# A Gut Feeling: Delusional Parasitosis

**DOI:** 10.1192/bjo.2025.10736

**Published:** 2025-06-20

**Authors:** Chris Joseph, Mario Lepore

**Affiliations:** South London and Maudsley NHS Foundation Trust, London, United Kingdom

## Abstract

**Aims:** Delusional parasitosis, first described by Karl Ekbom in the 1930s, is a rare psychiatric disorder characterised by a persistent, false belief of parasitic infestation. The condition is typically classified into three categories: primary, secondary and organic. Primary delusional parasitosis arises in the absence of any other psychiatric or medical condition, while secondary and organic forms are associated with underlying psychiatric disorders or organic diseases. Here, we present the case of a 50-year-old male with a history of crack cocaine use, previously unknown to mental health services, presenting to our drug treatment centre with delusions of infestation.

**Methods:** Mr A, a 50-year-old male with a 20-year history of crack cocaine use, was referred for psychiatric review by his keyworker after expressing unusual beliefs. He had been engaged in treatment for his substance use for the past year. During this period, he disclosed a persistent belief that he had contracted a parasitic infection in his gastrointestinal tract, which he attributed to consuming sashimi during a trip to Cambodia a decade ago. He described feeling worms moving within his abdomen, with heightened nocturnal activity that disrupted his sleep. His appetite was affected by fear of worsening the infestation, though no significant weight loss was noted. His mental state exam revealed no signs of thought disorder, additional delusions or perceptual disturbances. His cognitive function, social interactions, and self-care remained intact. Despite reassurance that repeated blood tests and abdominal ultrasound scans showed no abnormalities, his delusions persisted.

**Results:** Substance misuse, particularly with stimulants such as cocaine and amphetamine, is a well-established risk factor for delusional parasitosis. Chronic stimulant use can result in a dysregulated dopamine system, contributing to psychotic symptoms. In Mr A’s case, his long history of crack cocaine use is considered the primary contributing factor to his condition. While delusional parasitosis is typically associated with delusions of skin infestation, this case is notable for its gastrointestinal presentation, which is considerably rarer. Importantly, there was no indication that there were other contributory psychiatric or organic factors.

**Conclusion:** The management of delusional parasitosis requires a holistic, multidisciplinary approach. It is important for health professionals to address the patient’s beliefs with empathy to promote trust and encourage engagement. Addiction services continue to support Mr A’s efforts to reduce cocaine use, while mental health services have initiated antipsychotic treatment and are providing psychological therapy. Early indications suggest a positive response to this integrated treatment plan.

